# A Case Report on Myotendinous Junction

**DOI:** 10.7759/cureus.42233

**Published:** 2023-07-21

**Authors:** Aditya Kundu, Pooja Bhadoria

**Affiliations:** 1 Anatomy, All India Institute of Medical Sciences, Rishikesh, IND

**Keywords:** histology, tendon, muscle, myotendinous junction, mtj

## Abstract

Myotendinous junction is the transition zone between the muscle and its tendon. Hence, it is subject to immense stress within the muscle. In this study, it is found that muscles having a greater tensile have a more arranged myotendinous junction compared to muscles with lesser tensile strength. Cadaveric specimens - plantaris, gastrocnemius, and soleus have been histologically studied to study the same.

## Introduction

The myotendinous junction (MTJ) is a complex specialized region located at the muscle-tendon interface that represents the primary site of force transmission. It is the region responsible for the transmission of contractile force from muscle to tendon. In this study, the variation of myotendinous junction, due to the tension generated (on a day-to-day basis) is studied. As the myotendinous is a discontinuous and heterogenous area, it is prone to ruptures. Thus the junction should be made extremely strong and resistant to stress.

## Case presentation

**Figure 1:**
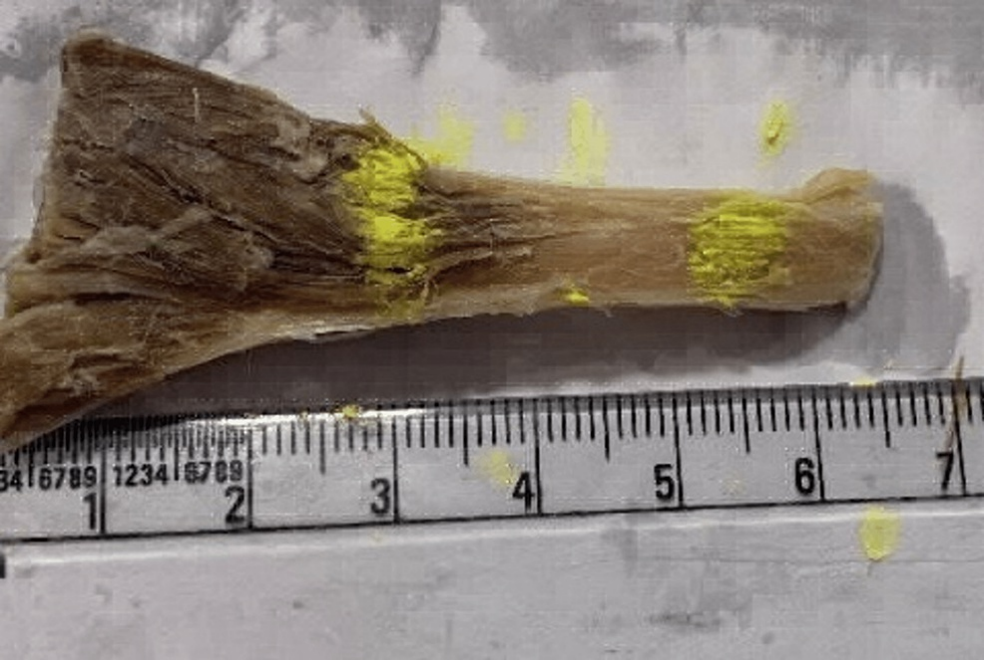
Gross myotendinous junction of soleus.

**Figure 2:**
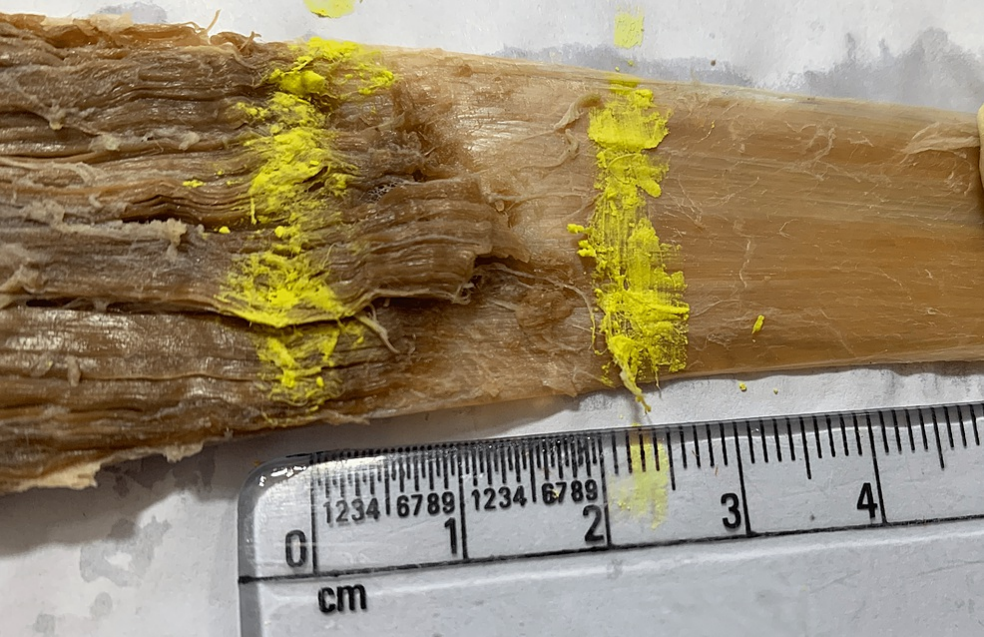
Gross myotendinous junction of gastrocnemius.

**Figure 3:**
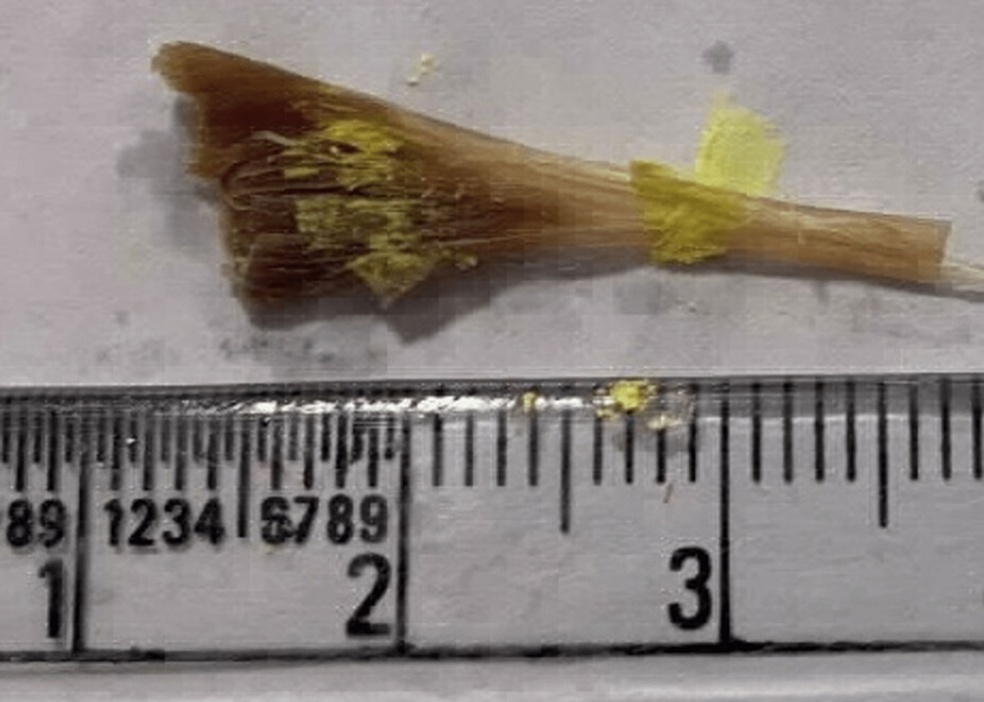
Gross myotendinous junction of plantaris.

This case was performed in the Department of Anatomy, All India Institute of Medical Sciences (AIIMS) Rishikesh, India. A lower limb of a cadaver was dissected and myotendinous junctions of three muscles namely - soleus (Figure 1), gastrocnemius (Figure 2), and plantaris were taken out (Figure 3). The tensile strength of a muscle depends majorly on its cross-sectional area. Thus, soleus is considered to be a high-tensile muscle whereas plantaris a low one. Gastrocnemius which comes between the two muscles when it comes to tensile stress was taken to establish continuity. The myotendinous junctions of each of the muscles were dissected out, processed, and stained with both the hematoxylin and eosin stain and the Masson's trichrome stain and observed under the microscope.

Soleus

**Figure 4:**
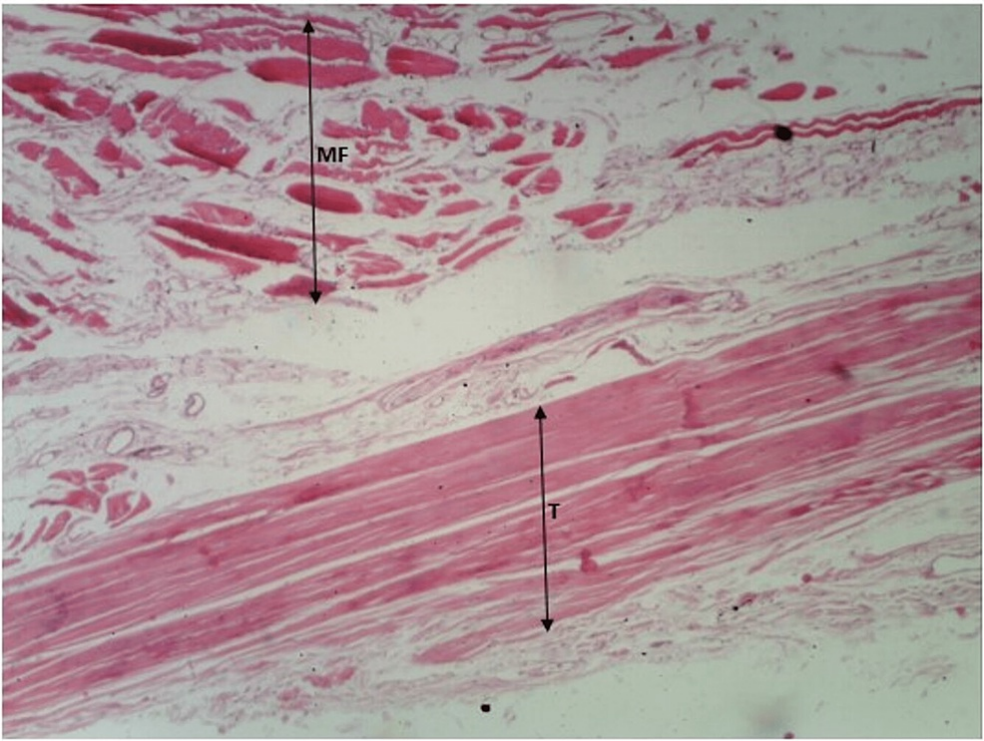
H&E staining of myotendinous junction of soleus. H&E: hematoxylin and eosin; MF: muscle fiber; T: tendon

**Figure 5:**
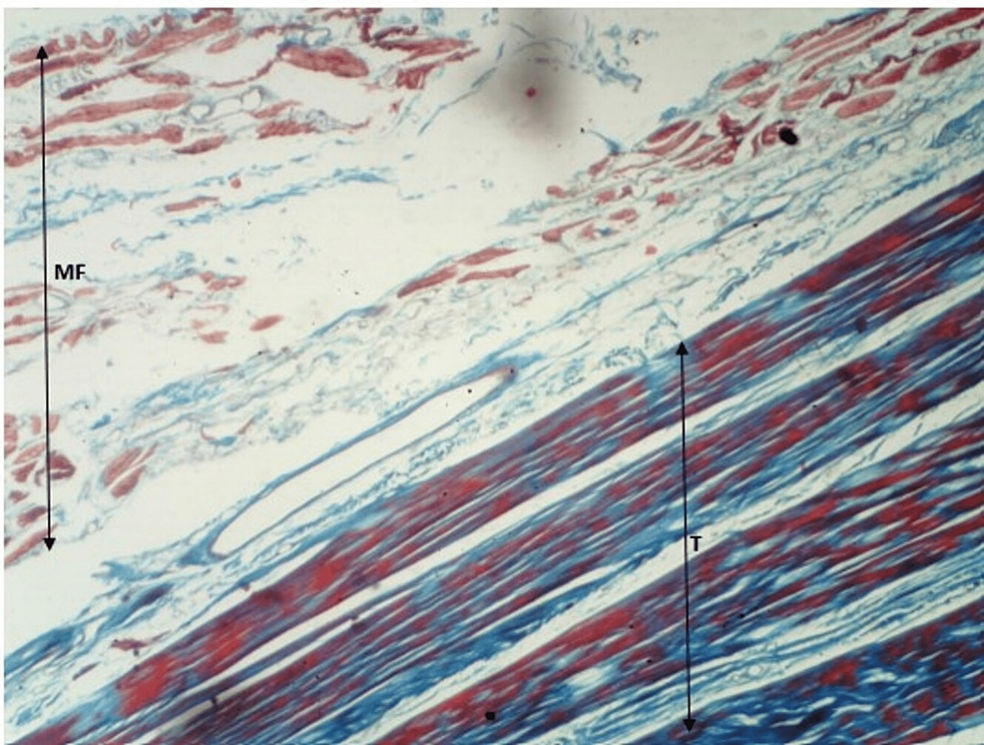
Masson's trichrome staining of myotendinous junction of soleus. MF: muscle fiber; T: tendon

As shown in Figure 4 and Figure 5, we can appreciate the ends of muscle fibers and tendons distinctly. Also, we can appreciate the myotendinous junction (the region between muscle fibers and tendon) clearly. The observations are consistent with both stains.

Plantaris

**Figure 6:**
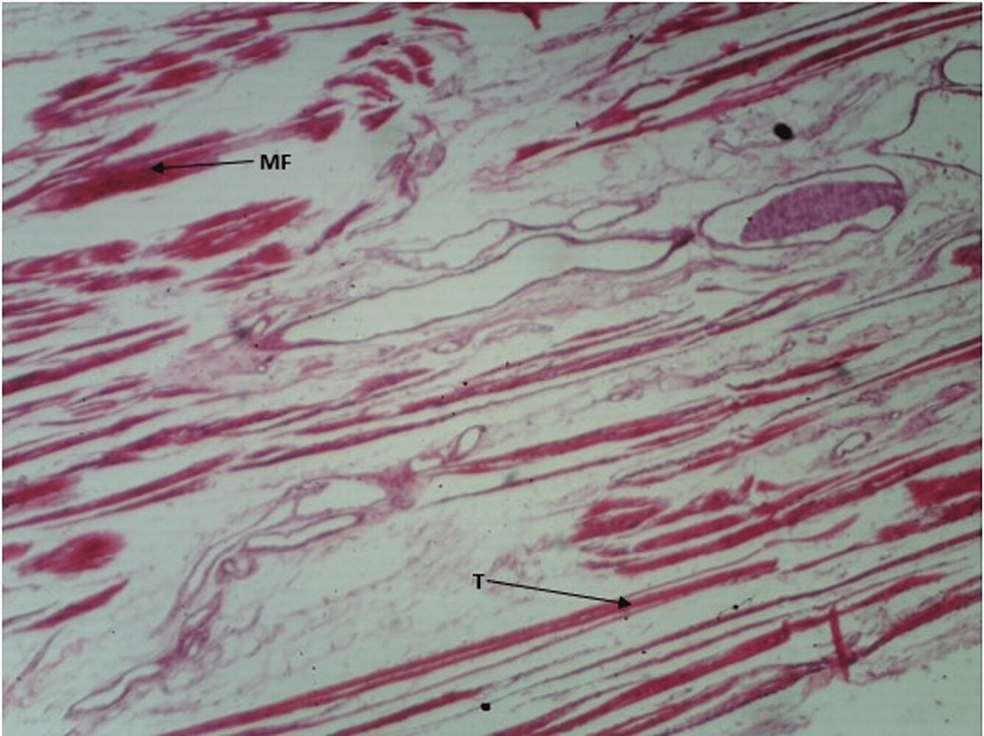
H&E staining of myotendinous junction of plantaris. H&E: hematoxylin and eosin; MF: muscle fiber; T: tendon

**Figure 7:**
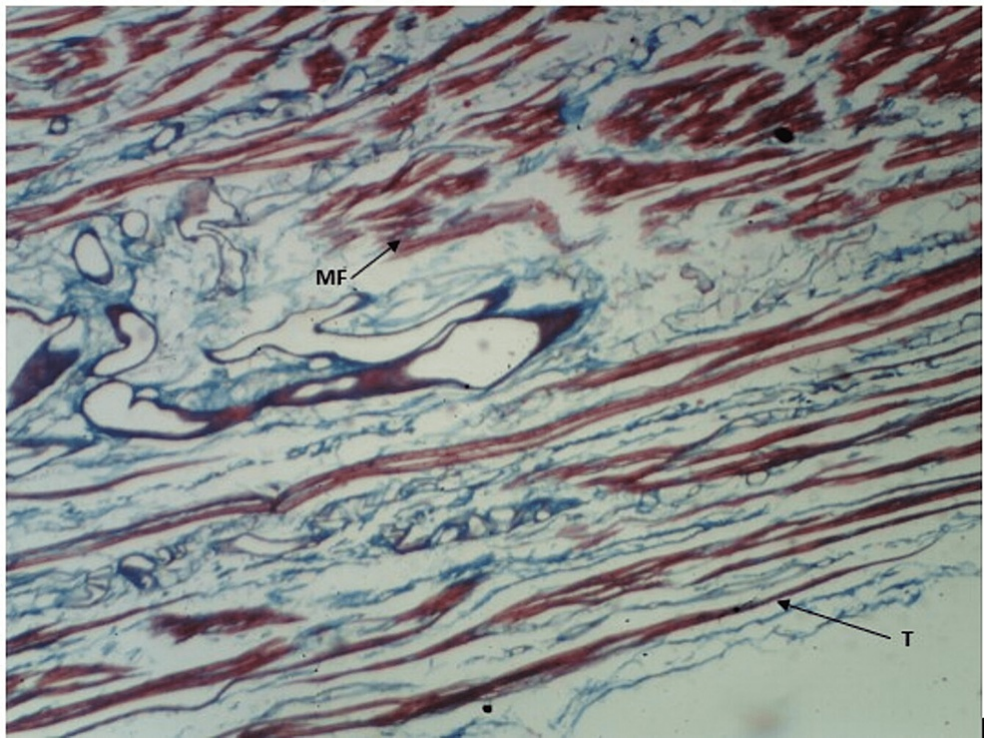
Masson's trichrome staining of myotendinous junction of plantaris. MF: muscle fiber; T: tendon

As shown in Figure 6 and Figure 7, we cannot appreciate the ends of muscle fibers and tendons distinctly. Also, we can appreciate that the myotendinous junction (the region between muscle fibers and tendons) is very diffusely arranged. The muscle fibers and tendons are visible between each other. The observations are consistent with both stains.

Gastrocnemius

**Figure 8:**
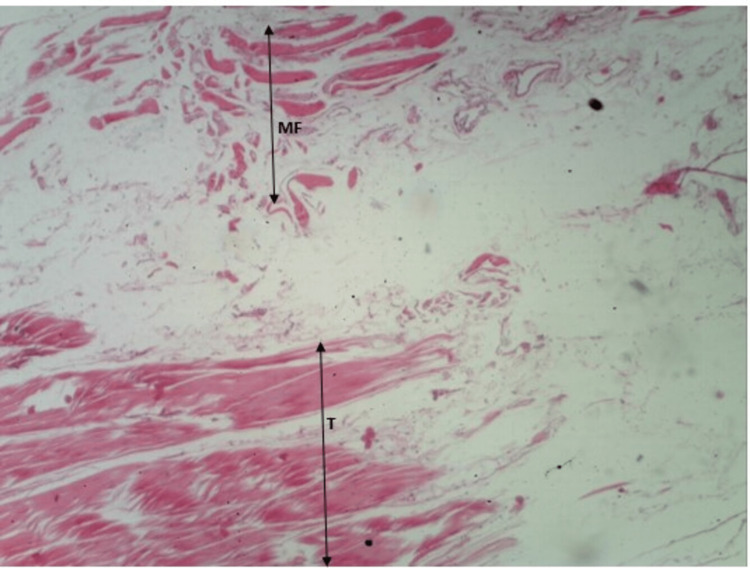
H&E staining of myotendinous junction of gastrocnemius. H&E: hematoxylin and eosin; MF: muscle fiber; T: tendon

**Figure 9:**
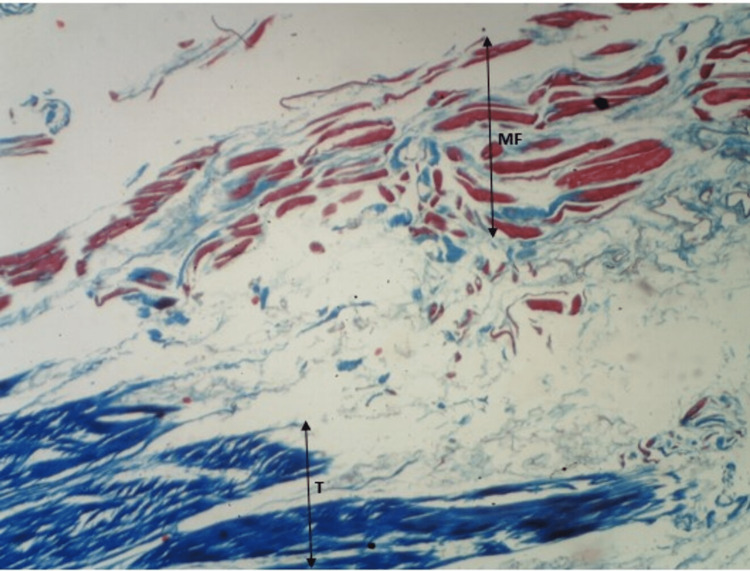
Masson's trichrome staining of myotendinous junction of gastrocnemius. MF: muscle fiber; T: tendon

As shown in Figure 8 and Figure 9, we can appreciate the ends of muscle fibers and tendons. Also, we can appreciate that the myotendinous junction (the region between muscle fibers and tendon) is more diffuse than soleus but sharper than plantaris. Very few muscle fibers and tendons are visible between each other. The observations are consistent with both stains.

## Discussion

The structural relationship between the cytoskeleton proteins and components of the extracellular matrix is responsible for the transmission of contractile force between the intracellular and extracellular muscles [1]. It consists of muscles and tendons which help in force generation and energetics during human movement [2]. The interaction between muscles and tendons allows effective force utilization and tendon elasticity in performing specific movements [3,4].

Exercise intensities lead to myotendinous junction undergoing certain morphological changes. These changes support the transfer of higher levels of tensile strength [5]. Automatic tracking of MTJ displacement can be seen in ultrasound images if sufficient knowledge about myotendinous junction and tendon tissue is present. The method targets the influence of non-tendinous components on the calculation of affine transform parameters over the effective MTJ region [6]. Chemical and signalling pathways involved in tissue differentiation and morphogenesis can be studied by studying the development of MTJ [7]. Miranda and Bureau concluded that the prevalence of supraspinatus myotendinous junction injuries was 0.47% [8]. The injuries exclusively were in the anterior part of the supraspinatus muscle and Taneja et al. established that rotator cuff myotendinous junction injuries affect mostly the infraspinatus and supraspinatus muscles, usually in a strain pattern and not in the region of tendon attachment [9]. Muscles and tendons have different embryological origins, but morphogenesis occurs in close spatial and temporal associations [10].

For amplifying the interactions between muscle and tendon, the MTJ is arranged in a fingerlike pattern where the tendon processes penetrate the muscle mass. The plasticity of the myotendinous junction, which appears at the morphological, structural, and functional levels, is due to different physiological and pathological stress [11]. Knudsen et al. observed that the tendon made ridge-like protrusions, which interdigitated with groove-like indentations in the muscle cell [12]. Finger-like processes help in the transmission of muscle contraction force to the tendon. Intracellularly, muscle failure is seen just proximal to the structurally defined MTJ, in the muscle cell body and not in the MTJ [13].

The peak torque after static stretching is related to a decrease in the muscle-tendon unit (MTU), muscle and tendon stiffness [14]. The ballistic stretching training program did not affect contractile elements and connective tissues (endomysium, perimysium, and epimysium). Also, the measurements of fascicle length and pennation angle during the range of motion (RoM) remained unchanged. A six-week ballistic stretching training program for the lower-leg muscle increases dorsiflexion RoM but does not affect muscle and tendon tissue [15]. During a concentric contraction, the involvement of the tendon depends on the range of motion used for the analysis [16]. The pathological and clinical variability was found to accompany the same genetic mutation, hence having a significant role for modifier genes in distal myopathy (MPD1) pathogenesis [17]. MRI can detect infraspinatus tears at the level of MTJ. Isolated infraspinatus damage at the MTJ can be detected by ultrasound too. Ultrasound can detect rupture of the main tendon skeleton which is located in the main body of the muscle. USG studies need to be considered in patients with rotator cuff tendon disease [18].

In this case, we have found that soleus, the muscle which is responsible for maximum tensile strength, has a sharp and well-developed muscle-tendon junction. Whereas, plantaris which is a spare muscle (lowest tensile strength) has a diffusely arranged muscle-tendon junction. Gastrocnemius which has intermediate tensile strength has its level of muscle-tendon junction arrangement between the other two.

## Conclusions

In this case, we have found that the muscle with greater tensile strength has a sharper MTJ while the muscle with lesser tensile strength has a diffused MTJ. The organization of the MTJ is found to be dependent on the tensile strength of the muscle. This observation is restricted to the study we performed. More research can be conducted to check its consistency and finally establish a hypothesis that is statistically relevant. This study will be useful to study sports injuries and normal muscle physiology. Very few studies were carried out in this field hence this study will stimulate many research work.
